# Knowledge, Awareness, and Attitudes Relating to the COVID-19 Pandemic Among Different Populations in Central China: Cross-Sectional Survey

**DOI:** 10.2196/22628

**Published:** 2020-10-15

**Authors:** Huifang Xu, Maria Jose Gonzalez Mendez, Lanwei Guo, Qiong Chen, Liyang Zheng, Peipei Chen, Xiaoqin Cao, Shuzheng Liu, Xibin Sun, Shaokai Zhang, Youlin Qiao

**Affiliations:** 1 Department of Cancer Epidemiology Affiliated Cancer Hospital of Zhengzhou University/Henan Cancer Hospital Zhengzhou China; 2 School of Public Health Dalian Medical University Dalian China; 3 Department of Epidemiology National Cancer Center/National Clinical Research Center for Cancer/Cancer Hospital Chinese Academy of Medical Sciences and Peking Union Medical College Beijing China

**Keywords:** COVID-19, knowledge, awareness, attitude

## Abstract

**Background:**

The COVID-19 pandemic has threatened the health systems of many countries worldwide. Several studies have suggested that the pandemic affects not only physical health but also all aspects of society. A lot of information has been reported about the disease since the beginning of the outbreak. For that reason, it is essential to investigate the attitudes and level of knowledge and awareness that different populations had regarding COVID-19 during the critical period of the outbreak.

**Objective:**

This study aimed to assess the knowledge and awareness of and attitudes toward the COVID-19 pandemic among different populations in Central China during the critical period of the outbreak.

**Methods:**

A cross-sectional web-based survey was conducted in Central China from February to March 2020. The study participants included three different populations: medical workers, students, and those with other occupations. In this study, a questionnaire was designed to collect information on the following four aspects: sociodemographic information, knowledge related to COVID-19, awareness of COVID-19, and attitude toward COVID-19. The chi-square test and Fisher test were used for comparison among groups. The level of significance was set at *P*<.05.

**Results:**

This study enrolled a total of 508 participants. Among them, there were 380 students (74.8%), 39 medical workers (7.7%), and 89 people with other occupations (17.5%). Most of the participants were female (n=272, 53.5%), lived in rural areas (n=258, 50.8%), and were single (n=423, 86.9%). The majority of the respondents had attended college (n=454, 89.4%). Most of the participants said they had heard about COVID-19 by January, and most of them looked for information on social media (Sina Weibo, 84.7%), and WeChat and QQ groups (74.2%). The participants showed an adequate level of knowledge about COVID-19 with no significant differences among the groups. However, medical workers demonstrated a slightly advanced knowledge in their responses to professional questions such as the potential susceptible population, possible host, treatment of COVID-19, and disease category. A higher proportion of medical workers (71.8%) and those in the other occupations group (52.8%) were highly concerned about the COVID-19 pandemic. More than 43% of the participants stated that the lockdown of their village/city had a significant impact on their lives. Nevertheless, the majority of respondents had an overall optimistic attitude toward the control of the disease (92.1% of students [n=350], 94.9% of medical workers [n=37], and 92.3% of those in other occupations [n=83]).

**Conclusions:**

All three groups reported an adequate background knowledge about COVID-19 but medical workers showed a slightly advanced knowledge in their responses to professional questions. Most of the participants were highly concerned about COVID-19 during the critical period of the outbreak. The majority of respondents declared that the village/city lockdown policy had a significant impact on their daily life but most of them held an optimistic attitude toward the control of COVID-19.

## Introduction

Coronavirus disease (COVID-19) rapidly spread around the globe and it has led to an economic crisis alongside a health care crisis in many countries and regions across the world [1]. It was declared a pandemic by the World Health Organization (WHO) on March 11, 2020, affecting 114 countries by that time [2]. Even today, COVID-19 is still a threat to the health systems of nearly 150 countries and regions, especially those dealing with an international health emergency for the first time. In China, authorities and citizens acted quickly and effectively to contain the spread of COVID-19. The measures taken included several lockdown policies and strict preventive measures across the country [3], offering a model of COVID-19 pandemic control to other countries and regions.

The increasing use of computers, tablets, and smartphones enables the rapid dissemination of information through the internet and social media, but such information lacks an effective guarantee of quality. As with previous epidemics such as Ebola or Zika virus disease, the internet has been used to spread misinformation regarding the COVID-19 pandemic [4]. It has been reported that social distancing promotes the spread of misinformation and has a profound impact on psychological well-being in vulnerable populations. Misinformation may lead to uncertainty, which is increased due to the novelty of the disease [5]. Therefore, it is essential to raise awareness and distribute accurate information about COVID-19 to avoid misinformation and decrease unnecessary stress in the population. Studies have described the effects that the pandemic can have on specific groups, such as medical workers [6], children and teenagers [7], older adults [8], and students [9]. In China, a study conducted by Yulan Lin and colleagues [10] determined the knowledge, attitudes, and anxiety levels of the general population in relation to the COVID-19 outbreak, as well as its impact on them. However, the knowledge- and attitude-related items in their study were limited to the symptoms and modes of transmission of COVID-19 and did not explore the differences between different population groups. To avoid the above limitations, this study was designed and conducted to evaluate the knowledge and awareness of as well as attitude toward COVID-19 of different populations groups in Central China during the critical period of the outbreak. This information might be necessary for policy makers to promote health education campaigns to better control the disease before the availability of a preventive vaccine. As there is no available vaccine or effective antiviral treatment against COVID-19, the active engagement of the population in preventive behaviors is necessary. For that reason, an adequate knowledge and high level of awareness of COVID-19, as well as an optimistic attitude, are crucial for controlling the disease.

## Methods

### Study Design and Participants

This study presents data from the Central China part of a population-based multicenter cross-sectional online survey, conducted in seven geographical regions across China (Northeast China, East China, South China, North China, Northwest China, Southwest China, and Central China) in February and March 2020. The anonymous survey was conducted via Wenjuanxing, an online crowdsourcing platform in mainland China that provides professional online surveys, voting, testing, and comments. The survey was developed to investigate the knowledge and awareness of and attitude toward the COVID-19 pandemic in different populations (medical workers, students, and those in other occupations). The survey contained items related to participants’ demographic background, knowledge, awareness, and attitudes.

The inclusion criterion was that the respondents were at least 18 years old when they filled out the survey. Thus, anyone that had the ability to complete this survey and met the age requirement was qualified to take part in the study. Respondents in this study were divided into three different population groups: students, medical workers, and those in other occupations.

Before starting the online questionnaire, a brief introduction was displayed to each participant, and electronic informed consent was obtained if they agreed to complete the questionnaire.

To confirm the quality of the online survey, two control items were included in the questionnaire. The first one was gender; although gender information was recorded initially, it appeared one more time in the questionnaire with confusing options. Another control item was a question about whether the influenza vaccine can prevent COVID-19. Two different descriptions were displayed, and the answers for the control items were supposed to match. Moreover, the members of the research team had been trained to ensure the quality of the questionnaire. Data cleaning and checking were done once the questionnaire was submitted.

### Sociodemographic Information

In the questionnaire, the following sociodemographic information was collected from all participants: gender, ethnicity, date of birth, marital status, household type, education background, monthly income, occupation, and smoking and drinking status.

In this study, respondents were identified as a current smoker or drinker if they reported being an active smoker or drinker.

### Knowledge and Awareness

The participants' knowledge was assessed using a series of questions related to COVID-19. The questions included the following items: “Did you hear about COVID-19?” “When did you hear about COVID-19?” “Do you agree that COVID-19 is the same as influenza virus?” “Do you agree that COVID-19 is the same as SARS?” “Do you agree that influenza vaccine can prevent COVID-19?” “Which level is the category and treatment of COVID-19 classified in China?” “What is the possible host for COVID-19?” “Which population group is susceptible to COVID-19?” “How long is the incubation period for COVID-19?” “Do you think that COVID-19 is contagious during the incubation period?” “Is there any effective treatment for COVID-19?” “When do you think that it is necessary to wear a mask during the COVID-19 pandemic?” and “Do you know that COVID-19 has been declared to be a Public Health Emergency of International Concern (PHEIC)?”

### Attitude Toward the COVID-19 Pandemic

In this section, the following items were designed to determine the participants’ attitude toward COVID-19: “What is your level of concern about COVID-19?” “How often do you check updates about COVID-19?” “What impact does the village/city lockdown policy have on your daily life?” “What is your attitude toward COVID-19 pandemic control?” and “Do you think that COVID-19 could be a global outbreak?”

### Ethical Considerations

This study protocol was approved by the Ethics Committee of Jining Medical College (JNMC-2020-KY-001).

### Statistical Analysis

We calculated counts and proportions for countable data in the questionnaire. Bar plots were used if necessary. The chi-square test was used to compare the differences in the countable data of groups. The Fisher test was applied to data that was not qualified for the chi-square test due to the small size of the sample. The differences between groups were considered statistically significant if the *P* value was <.05. All statistics were completed with SAS (Version 9.5; SAS Institute Inc).

## Results

### Sociodemographic Characteristics

Table 1 summarizes the sociodemographic characteristics of respondents by group. In this study, a total of 822 questionnaires were collected, 508 were used in analysis, and 314 were excluded due to the age limitation or inconsistencies in control item responses.

Of the 508 respondents, there were 380 students (74.8%), 39 medical workers (7.7%), and 89 people in other occupations (17.5%). The mean age was 24.1 years. The mean age for each group was 21.5 years for students, 29.6 years for medical workers, and 32.8 years for those in other occupations. In total, 272 participants (53.5%) were female. A slightly higher percentage of participants lived in rural areas (n=258, 50.8%) than urban areas (n=250, 49.2%), and most of them were single (n=423, 86.9%). The education level of the majority of the respondents was college and above (n=454, 89.4%). For monthly income, 58.7% had no income; this answer was especially high among students (76.9%). Most of the participants said they were not currently smoking (n=452, 89%) or drinking (n=344, 67.7%).

**Table 1:** Sociodemographic characteristic distributions of participants by occupation group.

Characteristics		Students (n=380)	Medical workers (n=39)	Other occupations (n=89)	Total (N=508)
Age (years), mean (SD)		21.5 (SD 3.48)	29.6 (SD 4.03)	32.8 (SD 9.95)	24.1 (SD 6.95)
**Gender, n (%)**					
	Male	190 (50.0)	12 (30.78)	34 (38.2)	236 (46.5)
	Female	190 (50.0)	27 (69.2)	55 (61.8)	272 (53.5)
**Ethnicity, n (%)**					
	Han	367 (96.6)	39 (100)	88 (98.9)	494 (97.2)
	Other	13 (3.4)	0 (0.0)	1 (1.1)	14 (2.8)
**Household type, n (%)**					
	Urban	161 (42.4)	30 (76.9)	59 (66.9)	250 (49.2)
	Rural	219 (57.6)	9 (23.1)	30 (33.7)	258 (50.8)
**Marital status, n (%)**					
	Single	372 (100)	16 (61.5)	35 (39.3)	423 (86.9)
	Married	0 (0.0)	8 (30.8)	51 (57.3)	59 (12.1)
	Others	0 (0.0)	2 (7.7)	3 (3.4)	5 (1.0)
**Education level, n (%)**					
	≤High school	2 (0.5)	5 (12.8)	47 (42.8)	54 (10.6)
	College and above	378 (99.5)	34 (87.2)	42 (47.2)	454 (89.4)
**Monthly income (¥), n (%)**					
	No income	292 (76.9)	0 (0.0)	6 (6.7)	298 (58.7)
	<4000 (<US $585)	86 (22.6)	8 (20.5)	23 (25.9)	117 (23.0)
	≥4000 (≥US $585)	2 (0.5)	31 (79.5)	60 (67.4)	93 (18.3)
**Current smoker, n (%)**					
	No	341 (89.7)	34 (87.2)	77 (86.5)	452 (89.0)
	Yes	39 (10.3)	5 (12.8)	12 (13.5)	56 (11.0)
**Current drinker, n (%)**					
	No	257 (67.6)	27 (69.2)	60 (67.4)	344 (67.7)
	Yes	123 (32.4)	12 (30.8)	29 (32.6)	164 (32.3)

### Knowledge and Awareness About COVID-19

Almost all of the respondents said they had heard about COVID-19 (99% of students, 100% of medical workers, and 98.9% of those in other occupations). A higher proportion of medical workers (n=18, 46.2%) and students (n=155, 40.8%) heard about COVID-19 by December 2019. However, a sizeable percentage of the respondents said they found out about COVID-19 between January 1 and 20, especially those in other occupations (52.8%) and students (49.5%).

Regarding the host of COVID-19, all medical workers reported that it was possibly a wild animal (eg, bats), and 98.7% of students (n=375) and 95.6% of those in other occupations (n=85) made the same choice. When the participants were asked about the populations most susceptible to COVID-19, 44.5% of the students (n=169) and 41.6% of those in other occupations (n=37) thought that middle-aged and older adults were more vulnerable. In contrast, 66.7% of medical workers answered that people of all ages were susceptible. The majority of the participants agreed that the incubation period of COVID-19 was between 1 and 14 days (86.5% to 92.3%), and over 98% of them agreed that the virus was contagious during the incubation period. Concerning treatment, most of the participants knew that there was no effective available treatment against COVID-19 (97.4% of medical workers [n=38] and 93.3% of those in other occupations [n=83]). In addition, 97.4% of medical workers (n=38) knew that COVID-19 was declared a PHEIC by the end of January, followed by students (n=319, 89%) and those in other occupations (n=78, 87.6%).

Regarding the use of masks, most of the participants agreed that it was necessary to wear a mask when going outside, with 90.5% (n=344), 87.2% (n=34), and 85.4% (n=76) of students, medical workers, and those in other occupations groups, respectively. This proportion was significantly higher than that of people that agreed to wear a mask in a crowded place.

As to the comparison between COVID-19 and other virus diseases, most of the students (n=321, 84.5%), those in the other occupations group (n=77, 86%), and all medical workers (n=39, 100%), knew that COVID-19 and influenza virus were not the same. Correspondingly, a high proportion of students (n=319, 84%), medical workers (n=37, 94.9%), and those in other occupations (n=76, 85.4%) answered that COVID-19 was different from severe acute respiratory syndrome (SARS). When participants were asked if the influenza vaccine can prevent COVID-19, a low proportion of students (n=33, 8.7%), medical workers (n=1, 2.6%), and those in other occupations (n=7, 7.9%) responded “Yes.”

Although the majority of information about COVID-19 was well known by the participants, with no significant differences observed among the groups, medical workers showed a significantly advanced knowledge in their responses to professional questions (Table 2).

**Figure 1:**
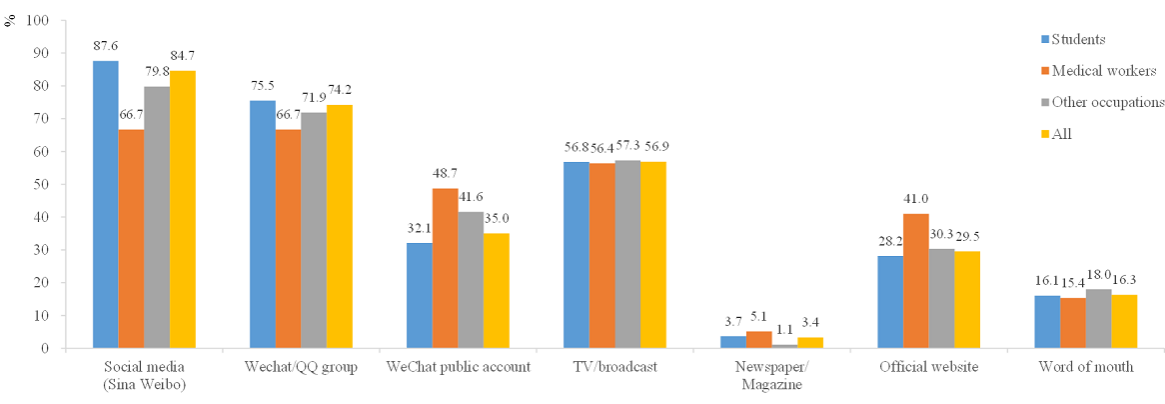
Ways by which participants obtained COVID-19 information, by occupation group.

Regarding the sources used to search for information about COVID-19, most of the participants obtained information from social media (Sina Weibo, 84.7%), as well as WeChat and QQ groups (74.7%). The least used sources of information for all three groups were newspapers and magazines (3.4%; Figure 1).

**Table 2:** Participants’ knowledge and awareness of COVID-19 by occupation group.

Questions and responses		Students, n (%)	Medical workers, n (%)	Other occupations, n (%)	*P* value
**Have you heard about COVID-19?**					>.99
	Yes	376 (99.0)	39 (100)	88 (98.9)	
	No	4 (1.0)	0 (0.0)	1 (1.1)	
**When did you hear about COVID-19?**					.72
	December 2019	155 (40.8)	18 (46.2)	30 (33.7)	
	January 1-20, 2020	188 (49.5)	17 (43.6)	47 (52.8)	
	January 21-23, 2020	31 (8.2)	4 (10.3)	10 (11.2)	
	January 24, 2020, and after	6 (1.6)	0 (0.0)	2 (2.3)	
**Do you know that COVID-19 has been declared to be a Public Health Emergency of International Concern?**					.06
	Yes	319 (89.0)	38 (97.4)	78 (87.6)	
	No	61 (16.1)	1 (2.6)	11 (12.4)	
**Do you agree that COVID-19 is the same as influenza virus?**					.03
	Yes	59 (15.5)	0 (0.0)	12 (14.0)	
	No	321 (84.5)	39 (100)	77 (86.0)	
**Do you agree that COVID-19 is the same as severe acute respiratory syndrome (SARS)?**					.19
	Yes	61 (16.1)	2 (5.1)	13 (14.6)	
	No	319 (84.0)	37 (94.9)	76 (85.4)	
**Do you agree that the influenza vaccine can prevent COVID-19?**					.41
	Yes	33 (8.7)	1 (2.6)	7 (7.9)	
	No	345 (91.3)	38 (97.4)	82 (92.1)	
**Which level is the category and treatment of COVID-19 classified in China?**					<.001
	Class B infectious disease and treated as Class A	189 (49.7)	33 (84.6)	34 (38.2)	
	Other	191 (50.3)	6 (15.4)	55 (61.8)	
**What is the possible host of COVID-19**					.09
	Wildlife (eg, bat)	375 (98.7)	39 (100.0)	85 (95.6)	
	Other	5 (1.3)	0 (0.0)	4 (4.4)	
**Which population group is susceptible to COVID-19?**					.02
	Middle-aged and older adults	169 (44.5)	7 (18.0)	37 (41.6)	
	Older adults and children	62 (16.3)	6 (15.4)	14 (15.7)	
	All ages	141 (37.1)	26 (66.7)	36 (40.4)	
	Young adults	8 (2.1)	0 (0.0)	2 (2.2)	
**How long is the incubation period of COVID-19?**					.63
	1-14 days	332 (87.4)	36 (92.3)	77 (86.5)	
	Other	48 (12.6)	3 (7.7)	12 (13.5)	
**Do you think that COVID-19 is contagious during the incubation period?**					.75
	Yes	374 (98.4)	39 (100)	89 (100)	
	No	6 (1.6)	0 (0.0)	0 (0.0)	
**Is there any effective treatment for COVID-19?**					.02
	Available	55 (14.5)	1 (2.6)	6 (6.7)	
	Unavailable	325 (85.5)	38 (97.4)	83 (93.3)	
**When do you think that it is necessary to wear a mask during the COVID-19 pandemic?**					.33
	When going outside	344 (90.5)	34 (87.2)	76 (85.4)	

### Attitude Toward the COVID-19 Pandemic

The three groups showed significant differences in their level of concern. A higher proportion of medical workers stated they were highly concerned (n=28, 71.8%), followed by those in other occupations (n=47, 52.8%) and students (n=149, 39.2%). The frequency of checking updates was also significantly different among the groups. Regarding the number of times per day participants reported checking for updates about COVID-19, 48.2% of students (n=183) checked once per day, as did 46.1% of those in other occupations (n=41). On the other hand, the proportion of medical workers that checked updates once per day (n=18, 46.2%) was slightly lower than the proportion that checked updates more than once per day (n=19, 48.7%). The proportion of participants that thought that COVID-19 would not be a global outbreak was 71.3% (n=271), 79.5% (n=31), and 69.7% (n=62) for students, medical workers, and those in other occupations, respectively.

Regarding the village/city lockdown policy, most of the participants from the three groups stated that the policy had a significant impact on their daily life (55.3% of students [n=210], 43.6% of medical workers [n=17], and 43.8% of those in other occupations [n=39]). Nevertheless, most of them had an optimistic attitude toward COVID-19 control (92.1% of students [n=350], 94.9% of medical workers [n=37], and 92.3% of those in other occupations [n=83]). Table 3 displays the attitude toward COVID-19 of participants by group.

**Table 3:** Participants’ attitude toward COVID-19 by occupation group.

Questions and responses		Students, n (%)	Medical workers, n (%)	Other occupations, n (%)	*P* value
**How concerned are you about COVID-19?**					.002
	I don't care	1 (0.3)	0 (0.0)	0 (0.0)	
	Low concern	56 (14.7)	2 (5.1)	7 (7.9)	
	Medium concern	174 (45.8)	9 (23.1)	36 (39.3)	
	High concern	149 (39.2)	28 (71.8)	47 (52.8)	
**How often do you check updates about COVID-19?**					.007
	More than once per day	104 (27.4)	19 (48.7)	33 (37.1)	
	Once per day	183 (48.2)	18 (46.2)	41 (46.1)	
	Less than once per day	93 (24.5)	2 (5.1)	15 (16.8)	
**What impact does the village/city lockdown policy have on your daily life?**					.27
	Minimal	59 (15.5)	8 (20.5)	17 (19.1)	
	Moderate	111 (29.2)	14 (35.9)	33 (37.1)	
	Significant	210 (55.3)	17 (43.6)	39 (43.8)	
**What is your attitude toward the control of the COVID-19 pandemic?**					>.99
	Optimistic	350 (92.1)	37 (94.9)	83 (92.3)	
	Neutral	9 (2.4)	0 (0.0)	2 (3.3)	
	Pessimistic	21 (5.5)	2 (5.1)	4 (4.5)	
**Do you think COVID-19 could be a global outbreak?**					.50
	Yes	109 (28.7)	8 (20.5)	27 (30.3)	
	No	271 (71.3)	31 (79.5)	62 (69.7)	

## Discussion

### Principal Findings

This study determined the knowledge and awareness of and attitude toward COVID-19 among students, medical workers, and those in other occupations during the critical period of the outbreak. It demonstrated that participants from different population groups had similar knowledge and awareness of COVID-19. However, medical workers performed significantly better on questions related to professional knowledge. It was confirmed that the lockdown policy had a substantial impact on the daily life of the analyzed groups. Nevertheless, most of the participants believed that COVID-19 would not be a global outbreak and they held an optimistic attitude regarding the control of the COVID-19 pandemic.

In this study, 314 respondents were excluded from the analysis as they did not answer the control items correctly. Of 314 unqualified participants, students accounted for 77.4% (n=226), while 17.5% (n=51) were in the other occupations group, and 5.1% (n=15) were medical workers. No significant differences were found in the occupation distribution between the qualified and unqualified groups. However, the mean age of the unqualified group was significantly lower than that of the qualified group because of the high proportion of students. The difference in distribution of ethnicity and marriage status was not statistically significant between the two groups. Thus, it can be inferred that the participants in the qualified group are representative.

Our study revealed that most of the participants had heard about COVID-19, and a higher proportion of them had heard about it by January, which corresponded with other studies that found that the peak of information seeking about COVID-19 occurred by the end of January in various countries [11,12]. It is worth noting that a certain proportion of medical workers and students had heard about the virus by the end of December 2019.

Regarding general knowledge about COVID-19, our findings showed that almost all participants knew that the host of the virus was probably a wild animal. In addition, most of the participants knew that COVID-19 was different from influenza and SARS, and also had a good understanding of the incubation time of COVID-19, as well as the most susceptible populations. Medical workers showed a more advanced knowledge in their responses to professional questions than the other two groups. Compared with another similar study conducted in India, the level of knowledge and awareness among the participants in our study is higher; most of the respondents in India were passably aware of the basic elements of the disease [13]. Findings similar to ours were reported in another study performed in China that demonstrated that most of the participants were knowledgeable about COVID-19 [14].

Participants of this study used social media (eg, Sina Weibo) most often to obtain information about COVID-19, as well as other apps (WeChat and QQ). Traditional media such as newspapers and magazines were used less often to get updates. Similar findings were found in other studies that analyzed where people searched for information about COVID-19 [15-17]. A possible explanation could be that social media apps have become popular and easily accessible compared with traditional media sources, thus speeding up the spread of information.

Regarding preventive measures, our study found that most of the participants would wear a mask when outside and in crowded places. However, there is controversial information about the effectiveness of wearing a mask among the general population [18]. In East Asia, especially in China, wearing a mask has been one of the main preventive measures recommended by the government since the beginning of the outbreak. This was possibly one of the main differences in response compared with other places in the world where the use of masks was not compulsory for the general population [19]. In fact, wearing a mask has contributed much to the effective control of COVID-19 in China. Furthermore, a wide range of countries and regions have recommended their citizens wear a mask if social distancing cannot be ensured.

Most of the study participants, especially medical workers, reported that they were highly concerned about COVID-19. Similarly, another web-based study conducted in China suggested that 97.1% of participants paid close attention to COVID-19 by checking updates more than once per day [20]. A possible reason for frequent update checking is the psychological stress associated with the pandemic that the public has experienced. Another study in China showed that more than 90% of the study population experienced concern and stress [21]. The high level of concern among medical workers could be due to their higher risk of exposure to COVID-19 and their fear of getting infected. A similar study performed in Henan, China, demonstrated that 85% of health care workers were afraid of being infected at work [22].

The lockdown policy has been an important preventive measure to deter the spread of COVID-19 in several countries [23,24], and it has been effective in China [25]. In our study, most of the respondents reported that the lockdown policy had a significant impact on their daily life. In this regard, other studies also confirmed that lockdown measures can have a substantial impact on the mental health of the population and affect people’s daily lives due to social distancing and decreased physical activity [26]. Moreover, another study that reviewed the impact of COVID-19 on university students showed that most of them were concerned about the effects that the outbreak would have on their academic performance. Most universities closed and classes were online [27]; this may be why students considered the impact of lockdown significant. Nevertheless, as a positive outcome, the lockdown policy also provided the opportunity and time for families to stay close, especially if they learned to cope with tensions that occurred due to changes in the family routine. However, several countries, including China, reported an increase in domestic violence during lockdown [28]. There was also a general concern about parenting during the lockdown and how families could deal with the stress of the crisis [29].

Despite being highly concerned about COVID-19, more than 90% of the respondents declared to have an overall optimistic attitude toward COVID-19 control. Similar findings were reported in another cross-sectional web-based study among health care workers globally, especially in Asia, where 78% of the participants held a positive perception of COVID-19 [30]. In contrast, a study performed in Uganda showed that only 21% of the participants had a good attitude toward the disease [31]. A possible cause of these differences in attitude toward the COVID-19 pandemic may be the social and economic gap among countries, which might influence the availability of medical services and sanitary conditions in houses and workplaces. The Human Development Index (HDI) might be an adequate measurement of this gap. In Asia, the HDI ranges from 0.647 to 0.866, while in Uganda it is 0.528. Similarly, more than half of the respondents said that COVID-19 could not become a global outbreak. This trend might be related to the low international incidence of cases when the survey was conducted.

This study evaluated the knowledge and awareness of and attitude toward COVID-19 among different populations in Central China, providing meaningful findings that might be useful for other countries and regions facing the COVID-19 pandemic. Nevertheless, limitations still exist. First, the sample size was small and unbalanced, especially for medical workers and the other occupations group, which might limit the generalizability of findings. Therefore, the interpretation of the results should be cautious. Second, the findings related to mental health were not included in the current analysis. Finally, the sample representing the target population could be limited to some extent because all participants came from the same geographical region.

### Conclusions

In general, the findings show that the participants have an adequate background knowledge of COVID-19 and hold an optimistic attitude toward the control of the disease. However, most of the participants were highly concerned about the pandemic and said that the lockdown policy had a significant impact on their daily life, suggesting a need for effective preventive measures to relieve emotional and psychological stress. Finally, this research offers essential evidence for COVID-19 pandemic control prior to the availability of a prophylactic vaccine. Further studies are still needed to identify the specific impact that the COVID-19 pandemic has had on the lives and mental health of the population.

## Abbreviations

HDI: Human Development Index
PHEIC: Public Health Emergency of International Concern
SARS: severe acute respiratory syndrome
WHO: World Health Organization
